# Collecting critically endangered cliff plants using a drone-based sampling manipulator

**DOI:** 10.1038/s41598-022-17679-x

**Published:** 2022-09-13

**Authors:** Hughes La Vigne, Guillaume Charron, Julien Rachiele-Tremblay, David Rancourt, Ben Nyberg, Alexis Lussier Desbiens

**Affiliations:** 1 Createk Design Lab, Interdisciplinary Institute for Technological Innovation, University of Sherbrooke, Sherbrooke, QC Canada; 2 National Tropical Botanical Garden, Kalāheo, HI USA; 3 Natural History Museum of Denmark, University of Copenhagen, Copenhagen, Denmark

**Keywords:** Mechanical engineering, Electrical and electronic engineering, Biodiversity, Conservation biology

## Abstract

Kauaʻi, an island within the Hawaiʻi archipelago, is home of a unique flora that contains 250 single-island endemic plant species. Threats have led to a significant population decrease where 97% of these plant species are now listed as endangered, critically endangered, or extinct. Vertical cliff habitats on Kauaʻi work as refugia to protect plants from their stressors. However, this habitat makes conservation work particularly difficult, forcing scientists, and botanists to use risky and time-consuming methods such as abseiling to access remote plant populations. Here we present the Mamba, the first aerial system capable of sampling plants on cliffs. This system is operated by two pilots and consists of an actively controlled platform suspended by a long cable under a lifting drone. Eleven otherwise inaccessible samples from five critically endangered species were collected during the first field trials on Kauaʻi Island. The samples are currently surviving in nurseries, demonstrating that the Mamba can be used to complete the conservation life cycle for organisms located in difficult areas, from location to collection, then cultivation and outplanting.

## Introduction

Plants are going extinct at rates of nearly 500 times what would be expected without human interference^1^. On Kauaʻi, one of the oldest of the Hawaiian high islands, where the field portion of this project was conducted, there are 250 single-island endemic plant species^2^. Most of these species are seeing significant population declines and habitat degradation, leading to 97%^3^ being listed as endangered, critically endangered or extinct on the 2021 International Union for Conservation of Nature (IUCN) Red List of Threatened Species^4^. Nearly 90 of these Kauaʻi endemics are extremely rare and have been reduced to total populations of less than 50 individuals, which puts them under the management of the Plant Extinction Prevention Program. The National Tropical Botanical Garden (NTBG) and partners have employed several methods to help prevent plant extinction, including robust ex-situ living collections, intra-situ plantings, in-situ restorations, and comprehensive seed banking, all of which rely on finding and collecting from these rare plants.

Threats to these species include feral non-native ungulates, invasive weed species and distinct environmental changes. Vertical cliff habitats on Kauaʻi work as refugia to protect plants from these stressors, while also providing additional benefits such as shade and wind protection^5^. To support conservation efforts, traditional botanical work is conducted by rappelling to plants from above, and occasionally climbing to them from below^6^. In Hawaiʻi alone, these techniques have led to at least 12 new species descriptions^7,8^. While effective, this method does have limitations in reach from above (e.g., rope length), and survey width, which is limited to just a few meters in either horizontal direction of the rope. In addition, rappelling work is inherently dangerous and time-consuming.

Drones, also called Unmanned Aerial Vehicles (UAV), have proven to be valuable tools in discovery, inventory, and mapping of rare cliff plants^9^. Due to the difficulty of on-the-ground survey of cliffs, drone have been deployed across a range of environments leading to discovery of unknown populations. Even with the significant increase of the total number of known individuals through drone surveys, many species remain some of the rarest plants in the world, including *Hibiscadelphus woodii*, *Lysimachia scopulensis* and *Euphorbia elaenoriae*. These tools have increased our understanding of overall distribution, habitat requirements and associated species. However, many of these plant populations occur in areas that are completely inaccessible to traditional botanical sampling methods.

Aside from common imagery applications, drones have been used recently to interact directly with their environment using manipulators^10–12^ specially designed for applications like infrastructure inspection^10–16^ and in-contact power line inspection^17–19^. The field of biodiversity conservation and monitoring also offers opportunities for aerial manipulation. For example, specialized aerial manipulators can now be used to collect samples from the top of trees^20,21^, as well as samples from the side of trees^22^. These systems make it possible to reach samples that were previously difficult to reach or completely unreachable with traditional techniques (e.g., climbing, pole pruners). Likewise, a specialized aerial manipulator that could sample specific parts of plants on cliffs (e.g., branches, scion, flower, seeds, fruits) would support the conservation efforts in Hawaiʻi.

**Figure 1:**
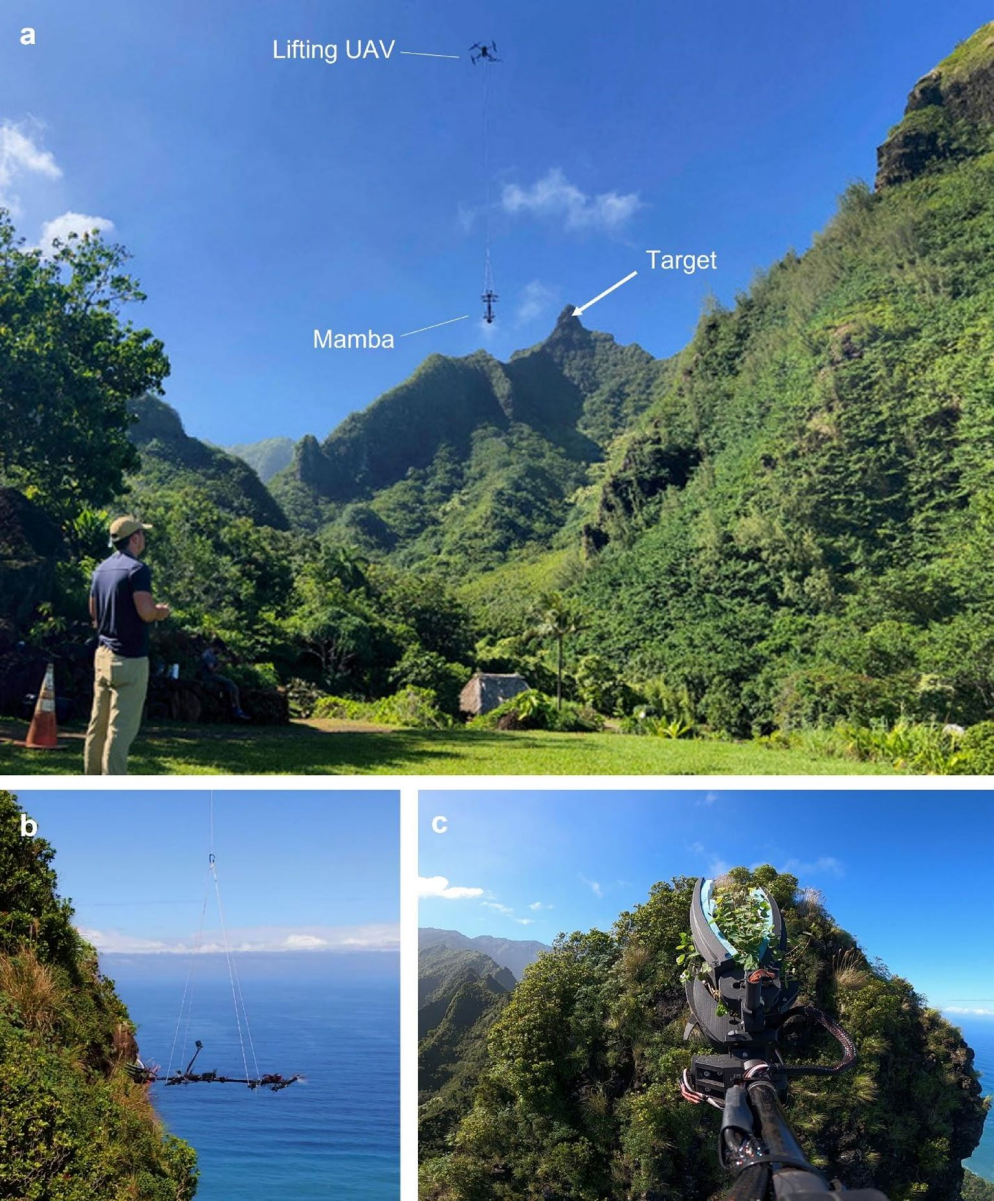
Aerial photos of the Mamba system during a mission at Limahuli Garden, Kauai, to collect *Euphorbia elaenoriae*. (**a**) Takeoff and travel to the target, located 1 km away from the base station. (**b**) Final approach on the target using the Mamba control system. (**c**) View as seen from the pilot from a first-person view camera with the sample secured in the tool.

Consequently, we present the Mamba, the first aerial system capable of sampling plants on cliffs. As shown in Fig. 1, this system consists of an actively controlled platform suspended by a long cable under a lifting drone. This decoupling between the suspended platform and the lifting drone allows the suspended platform to move quickly and precisely in the windy environments found around cliffs, while also being tolerant to the interaction forces generated during sampling. This also allows the lifting drone to remain safely away from the cliffs while plant material is being collected. Given the diversity of the target species, their rarity, and the unique environment of each sampling mission, the Mamba is teleoperated and equipped with an active wrist. The Mamba has demonstrated successful operations in its first field tests around Kauaʻi Island. The samples collected by the Mamba include 11 otherwise inaccessible samples from 5 species listed as critically endangered. Samples were often collected in less than 10 min of total flight time from cliffs that were more than 1 km away, in winds gusts of 13 to 37 km/h as shown in the Supplementary Video. Samples were vouchered and deposited at the Pacific Tropical Botanical Garden Herbarium and plant material was transferred to the NTBG nursery. The collections will help augment genetic diversity of existing ex-situ collections and represent the first time each of these sub-populations has been established ex-situ.

## Results

**Figure 2:**
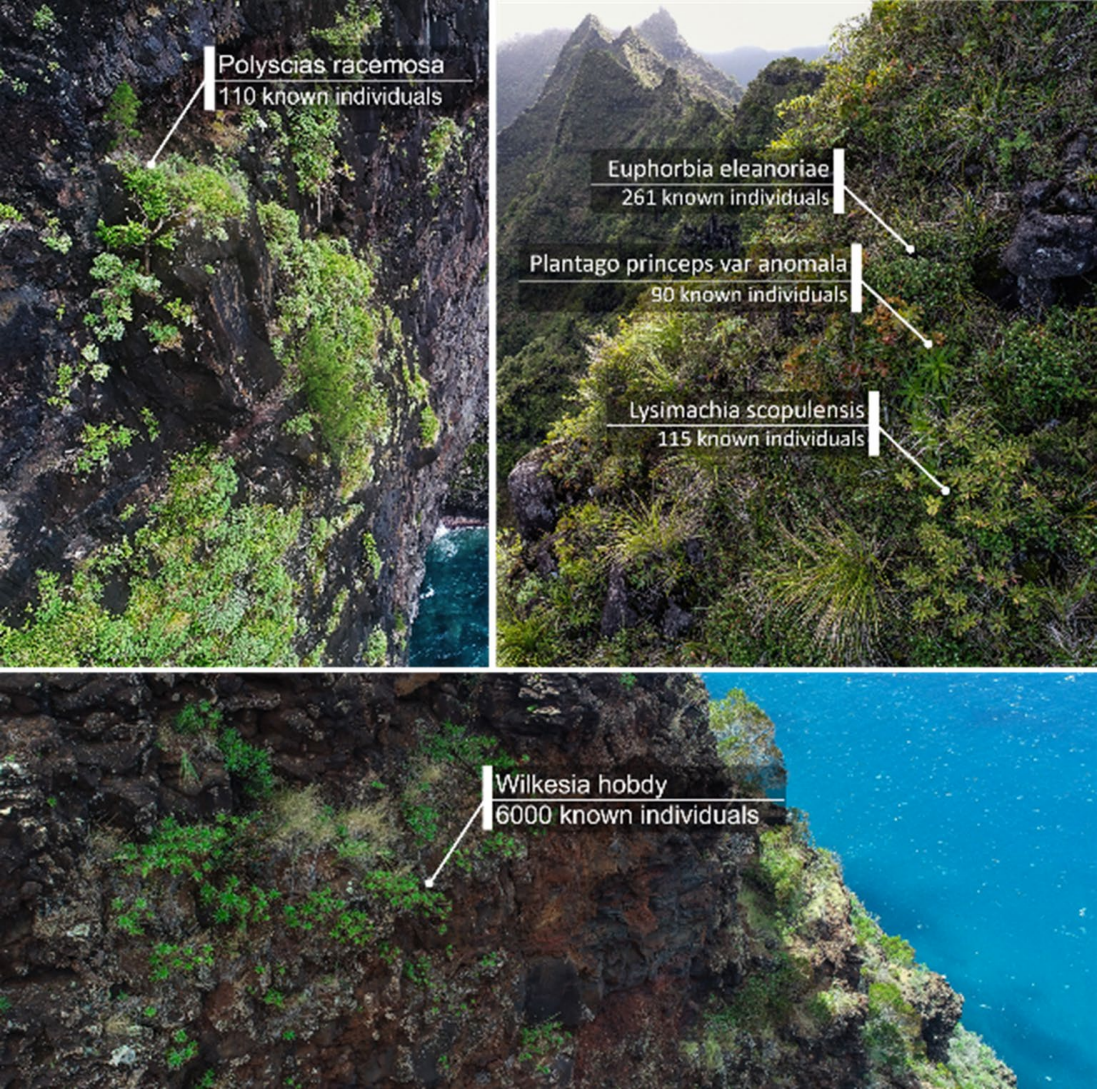
Examples of the cliff habitats of some critically endangered species on the Kauaʻi Island along with the count of known individuals as of February 2022.

Cliffs present a unique flora that has been little studied until now mainly because of the inherent difficulties to access this unique environment, as shown in Fig. 2. The techniques currently used to access plants on steep slopes and cliffs (e.g., abseiling, helicopter) are generally dangerous, costly and time consuming. Using a small aerial manipulator to sample plants on the cliffs can represent many advantages, including safety and portability, as well as the capability of reaching otherwise inaccessible locations easily, quickly and at low cost.

However, several technical challenges make it difficult to develop suitable aerial manipulators for this task. Indeed, the sampling of plants on cliffs necessarily leads to significant collision risks, as well as contact forces and moments during sampling that can destabilize the drone. The samples collected would also need to be accessed from the side of the aerial platform^22^. Any weight (e.g., sampling tool, collected samples) located horizontally away from the center of mass of the drone creates large additional demands on the propulsion system of most drones. To collect specific plant parts in windy conditions (e.g., scion, flowers, seeds, etc.), precise and fast motion is required even in degraded Global Navigation Satellite System (GNSS) coverage near the cliffs. The great diversity of plant species and morphology found on cliffs, as well as the variety of targeted sections of plant, also represent a major design challenge. Finally, to maximize the adoption of this tool, it is also desirable that scientists with minimal training could use this platform. The next sections describe how these challenges were addressed through the development of the Mamba.

### Suspended sampling platform

There are a multitude of configurations that could have been explored to sample plants on cliffs. Some drones have manipulators rigidly attached to their structure^20,23^. However, these manipulators tend to have a limited reach to keep the center of mass within the propeller footprint and to minimize the inertia of the system. This could result in a high collision risk with the propellers in the uneven terrain found on cliffs. The contact forces created during the sampling operation also generate destabilizing moments through manipulators rigidly attached to the drone. To address these challenges, concepts involving a compliant manipulator operated from specialized drones were also explored^10^. Alternatively, some aerial manipulators were also passively suspended under the drone through a long rod^21,24^. This keeps the drone above potential obstacles within the environment, significantly reducing the operator's mental demand and stress while also reducing the disturbances transmitted to the drone to a downward force aligned with the rod and yaw torque. To maintain these advantages while providing better precision, some projects have developed cable suspended platforms equipped with thrusters^25,26^. As these platforms do not have to counter gravity, the thrusters can be positioned to fight external disturbances more efficiently (e.g., wind, contact forces, drone movements). Existing systems however only stabilize the suspended platform close to its equilibrium point.

**Figure 3:**
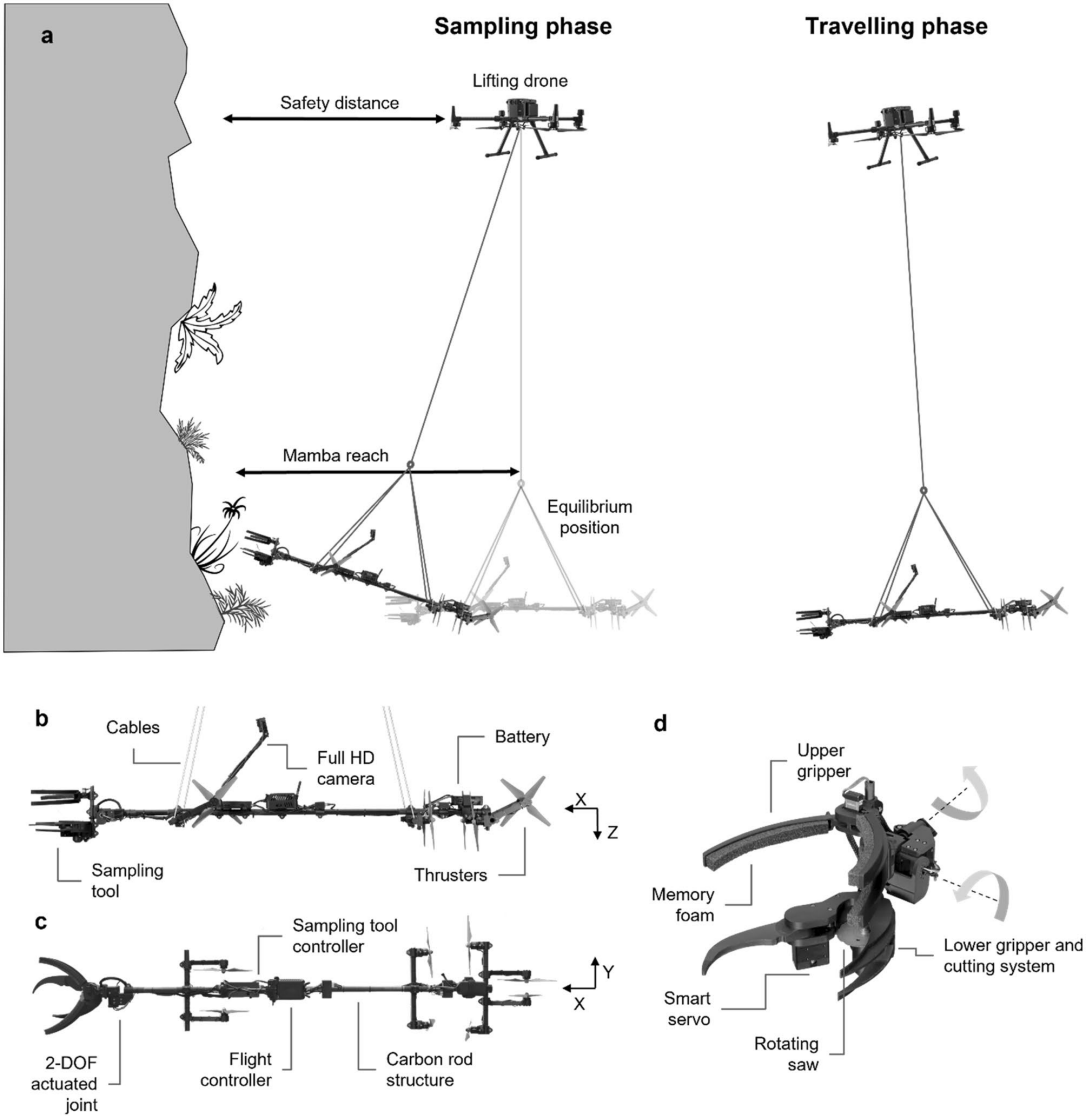
(**a**) General concept of the Mamba and lifting drone during transit and sampling on cliffs. (**b**) Side view of the Mamba showing the components and cable installations. (**c**) Top view showing the antagonist thrusters configuration. (**d**) Close-up of the sampling tool and 2 degrees of freedom (DOF) wrist specifically designed to sample small fragile plants.

The chosen concept for the Mamba, illustrated at Fig. 3, consists of a suspended platform that can stabilize itself far from its natural equilibrium to provide a large workspace. The lifting drone in this system stays safely away and above from steep cliff faces, while supporting the platform and providing rough positioning in space through better GNSS coverage. The platform is suspended 10 m below the lifting drone using four attachment points to prevent pitch and roll motions. The cable also acts as a low pass filter, isolating the platform from the fast drone movements required to fight wind disturbances. The suspended platform design can then focus on fast and precise positioning, while also being tolerant to contacts during sampling. To do so, four pairs of bidirectional actuators are used to control the motion in the plane of the pendulum (i.e., x and y translation, as well as yaw). Two pairs of actuators are installed in the x-direction to provide sufficient force to reach plants as far as 4 m from the equilibrium position. This corresponds to roughly 3.3 m from the tip of the lifting drone’s propellers.

Since the Mamba is self-powered and has its own communication system, the lifting drone function is simply to lift the platform and hold it in place. This made it possible to select amongst the many commercially available products to accelerate the development of the Mamba. The DJI M300 was chosen as it comes equipped with a 360° optical obstacle avoidance vision system, an IP45 rating, and a flight time of 20 min with the Mamba attached (3.3 kg). It also advertised a four constellation GNSS receiver for better coverage around buildings, structures, and cliffs.

### Precise control in winds

Winds under 20 km/h represent a gentle breeze on the Beaufort scale. At this level, the wind only moves the leaves, and not the branches, which allows for ideal sampling conditions. According to historical weather data from 2020, daily maximum winds are less than 20 km/h for 40 to 70% of the year, depending on the exact location on Kauaʻi Island (i.e., Lihuʻe International airport, as reported by the National Oceanic and Atmospheric Administration, and the Makaha Ridge Weather Station, as reported in the MesoWest database). This also implies that Kauaʻi experiences stronger winds on certain days which would make precise sampling difficult. Wind conditions are also more challenging near cliff faces, with increased turbulence and vertical airflow along the cliff.

To allow operations on most days, while providing precise positioning and fast rejection of wind disturbances, the actuators of the Mamba are oriented in the horizontal plane. This allows the actuator forces to directly affect the motion of the suspended platform. Each actuator of the Mamba consists of a pair of brushless DC motors and 23 cm propellers capable of producing 7 N of force. The motors are installed in opposite directions, are always idling at their minimum rotation speed, and are commanded to only create force in their preferred direction. This antagonistic configuration avoids the low-velocity dead zone of a brushless motor during thrust reversal. This makes it possible to quickly revert the direction of the thrust and nearly triples the bandwidth of the actuators to approximately 2.5 Hz^27^. This configuration, however, comes at the expense of added mass and components.

The Mamba is equipped with a flight controller that includes a control system, and a state estimator. To avoid degraded GNSS coverage issues, the state estimator only uses data from a high accuracy inertial measurement unit (IMU) to estimate the attitude of the platform. This provides the relative position of the platform with respect to the drone and is sufficient for teleoperation. Three separated proportional-derivative controllers are used for each of the DOF controlled by the actuators. This control system also provides attitude-hold assistance (i.e., pitch and roll, which correspond to x and y displacements, as well as yaw). This implies that if the user does not send any commands, the suspended platform maintains its current state.

**Figure 4:**
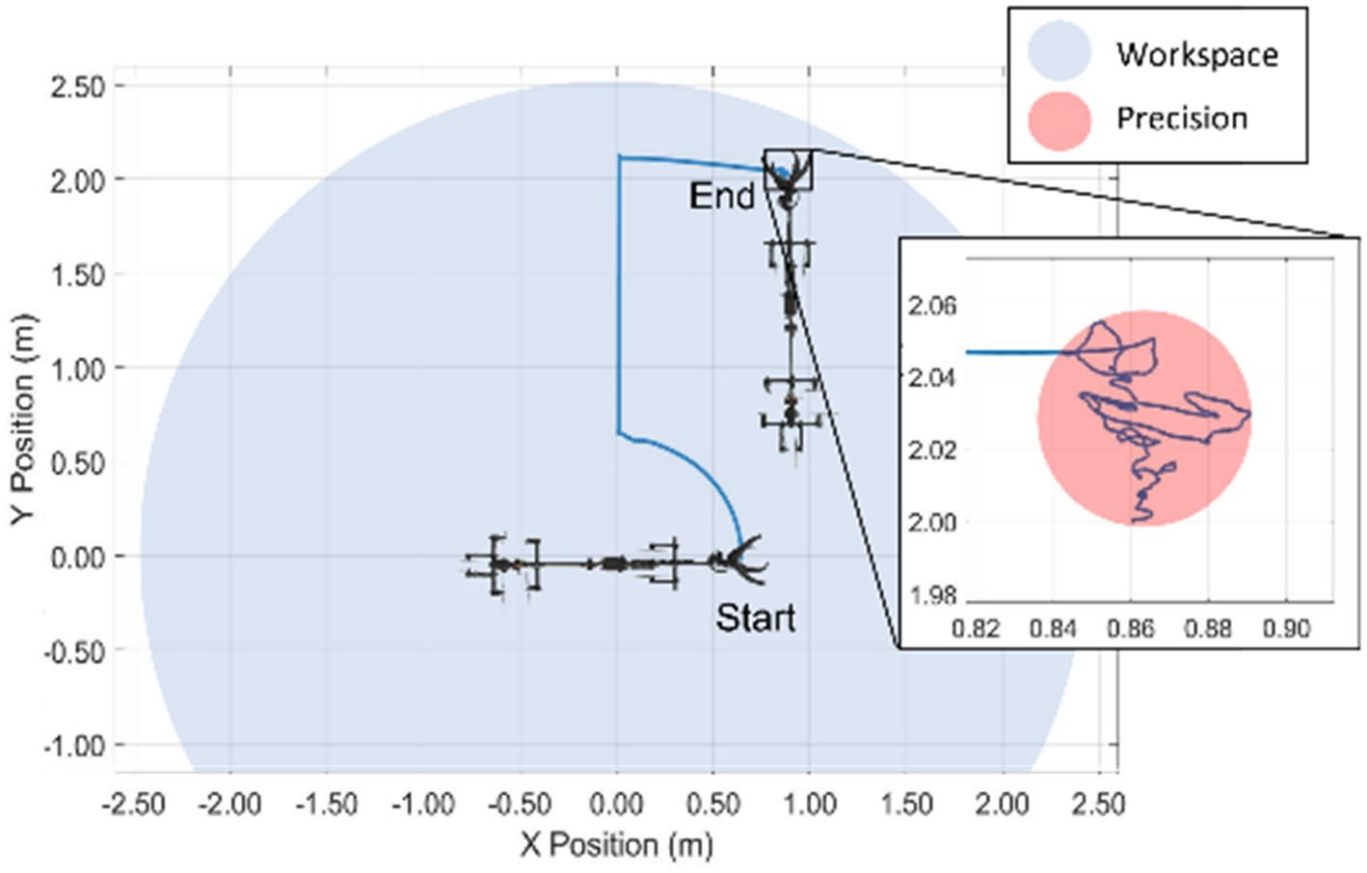
Representative motion of the sampling tool within its workspace based only on feedback from a high accuracy IMU and recorded using a motion capture system. The natural equilibrium point is at (0,0). The experiment starts with a 90° rotation around the z axis, followed by a forward movement along the x-axis of the Mamba and a lateral movement along its y-axis. The system then maintains this position for 30 s without any user inputs. Produced in MATLAB R2021a.

Figure 4 illustrates the stabilization accuracy of the Mamba when moving along a representative trajectory when suspended indoors from a 5.7 m cable (limited by ceiling height). This experiment confirmed that the sampling tool can maintain a position at a horizontal reach of 2.25 m with a precision of about 5 cm for 30 s. As the horizontal reach and precision are limited by the cable angular displacements (e.g., component of weight acting on the pendulum, IMU angular resolution), the resulting workspace when operating with a 10 m long cable would reach a radius of 4 m with a positioning accuracy of about 9 cm. To account for potential external disturbances like wind, the sampling tool was designed with an opening of 15 cm. This creates some margin for the pilot to align the target with the sampling mechanism. Field trials detailed below demonstrated that the Mamba actuators and controller could maintain a sufficiently stable position to sample plants in winds During the sampling phase, wind speed averaged 15.7 km/h with a standard deviation of 6.8 km/h, while wind gusts reached an average of 20.1 km/h with a standard deviation of 6.5 km/h. The maximum average wind speed recorded during sampling was 28 km/h with gusts up to 37 km/h. This represents a lower bound of the system performance, as no failure resulted from the wind conditions experienced during the trials. The a ttached Supplementary Video also demonstrates the stability of the system.

### Teleoperated sampling of cliffs habitats

Plants growing on Kauaʻi cliffs exhibit a wide morphological variety. For this project, targets ranged from small herbaceous plants such as *Euphorbia eleanoriae (plants* < *40 cm)*, to the multi-headed sub-shrub *Wilkesia hobdyi (plants* < *1 m)*, the larger shrub and tree form of *Hibiscadelphus distans (shrubs up to 5 m)* and even the full-sized tree *Flueggea neowawraea (trees up to 30 m)*. To accommodate these requirements, a specialized version of the DeLeaves’ tree sampling system (http://www.outreachrobotics.com) has been developed as shown in Fig. 3. This system includes a rotating saw and two grippers to grasp and hold the sample. The new system has been reduced in size to collect smaller plants. The sampling mechanism was designed to sample small parts from herbaceous plants and shrubs. Accordingly, the two grippers can securely hold branches up to 200 g. The length of the sample collected is limited to under 50 cm, depending on the sampling mechanism orientation. The distance between the two grippers is adjustable, from 9 to 15 cm, to accommodate different sample lengths. The cutting system consists of a brushless motor and a 77 mm diameter saw blade. The cutting system can cut a branch with a diameter up to 15 mm. The full cutting sequence is automated and takes less than 2.6 s, almost 3× faster than the DeLeaves sampler. This reduces disturbances on the suspended platform and allows easier sampling of small plants moving in winds. With the push of a button, the saw is accelerated. Then, the lower robotic gripper closes rapidly to capture the sample, before slowing down to bring the target sample into the saw at a controlled speed to prevent jamming. During that time, the upper gripper holds the sample in place. The upper gripper includes urethane foam covered with emery tape to conform to different sample shapes and increase grip on smooth stems. To align the cutting mechanism with samples growing in all directions from the cliff faces, a 2 DOFs actuated wrist is used to rotate the gripper in pitch and roll. The pitch range of motion goes from − 90° to 90° from vertical, while the roll range of motion goes from − 90° to 180°.

**Figure 5:**
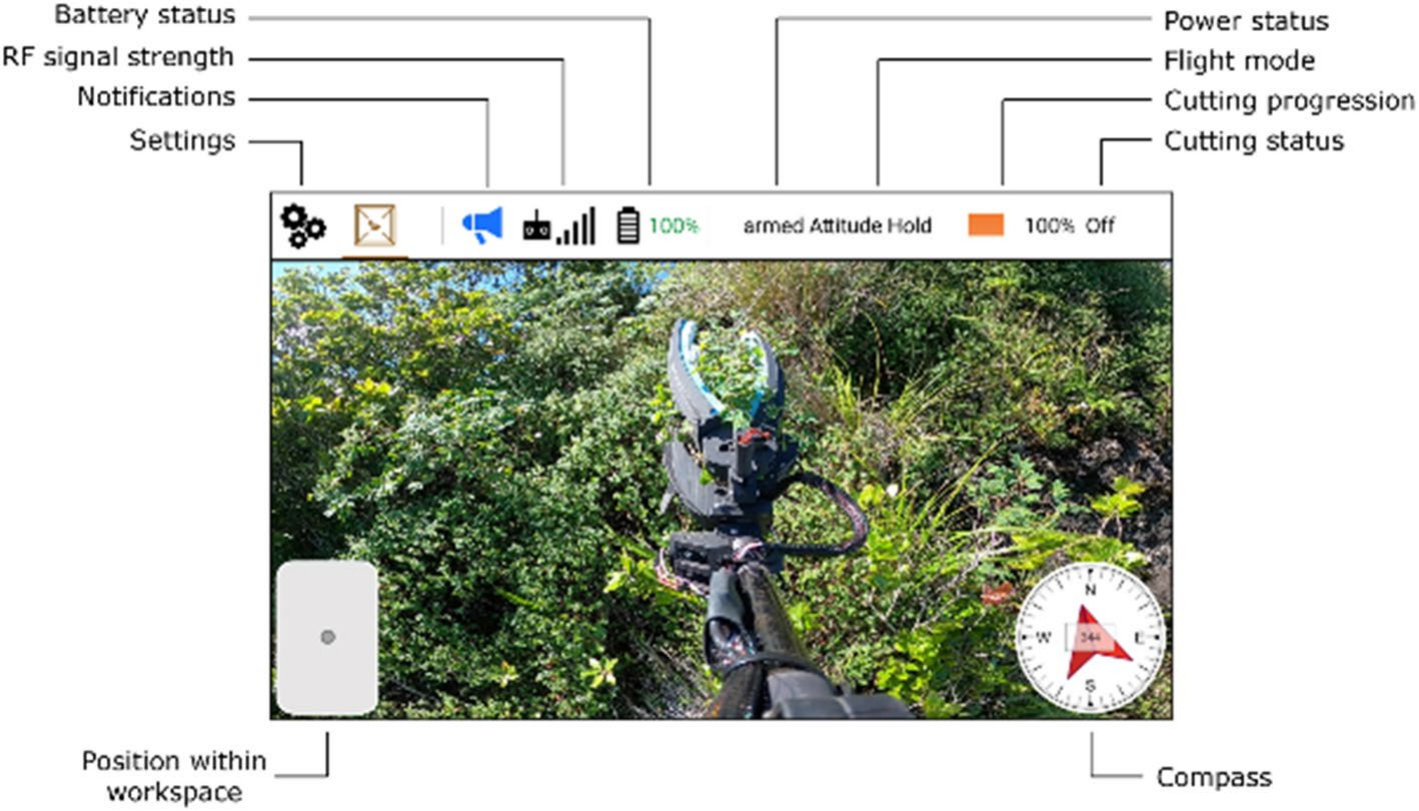
Overview of the user interface on the remote controller during the sampling of a *Euphorbia eleanoriae*. The Mamba position within workspace allows the operator to understand where the suspended platform is located with respect to the lifting drone. This insight allows the Mamba operator, and the lifting drone pilot to adjust the drone position if the target is located outside the sampling tool workspace.

To allow for greater adaptability to each unique sampling mission, and because the samples of interest are rare and of high importance, it was decided that the Mamba would be guided by an operator through teleoperation. The precise local positioning and augmented stability described earlier, coupled with a human operator, also reduces the needs for global positioning through sophisticated perception or GNSS. To allow target identification and teleoperation, the system is equipped with a high-definition camera and a video transmitter offering a first-person view during the sampling process. The remote controller is run with a custom software package based on QGroundControl^28^ which offers several status indicators as shown in Fig. 5.

### Field trials

Field trials were conducted in October 2021. Performance validation and pilot training was performed on common species before targeting rare plants. A ll collections were made under appropriate permits for threatened and endangered plants. Bio sanitation of the cutting mechanism was completed between each collection event using 70% isopropyl alcohol to prevent spread of potential contaminants, fungi, or diseases.

Prior to the field trials, the NTBG drone survey program, with the help of the Plant Extinction Prevention Program, located and prioritized targets of interest. Once on site with the Mamba, a first reconnaissance flight was carried out using a small drone to allow botanists to confirm the specific samples to target, while also allowing the aerial manipulator operators to fully understand the environment in which the sampling mission would take place. After the reconnaissance flight was completed, the drone- Mamba system was deployed to the location of the target plant. Deployment of the system on location typically takes less than 10 min. As the targeted environments have many natural obstacles (e.g., mountains, canyons, dense vegetation, overhanging parts of the cliff), operations carry the risk of degraded GNSS signals and unseen obstacles. To mitigate these risks, the lifting drone was manually controlled for all operations with the help of the DJI M300 optical obstacle avoidance system. In the event of entanglement, there is a Mamba emergency release to prevent damage to the plant.

Operation of the drone-Mamba requires two users. The first user controls the lifting drone to move towards the target location and position the desired sample within the Mamba workspace. The second user then maneuvers the Mamba to position the plant inside its sampling tool and activates the cutting system to collect the sample. Once collected, the wrist can be reoriented to secure the sample. When multiple flights are planned during the same field day, two battery sets are used and recharged with a generator to allow for continuous operations.

**Figure 6:**
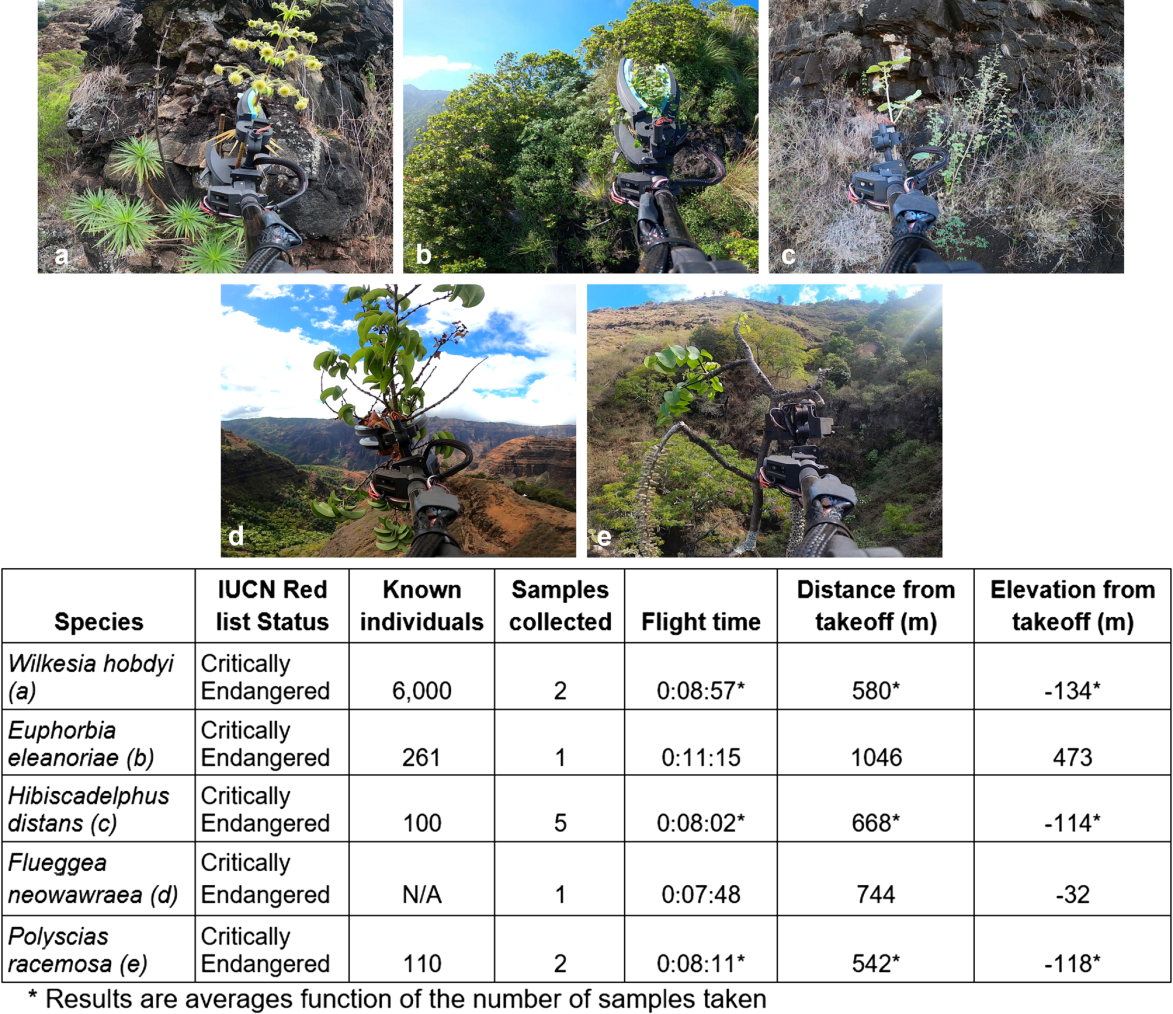
List of critically endangered plant species collected during the field trials. Flight time and the distances traveled by drone from the take-off point are also indicated. Note that the sample in picture (**e**) is held upside down in the sampling mechanism.

The Mamba was successful in collecting 11 specimens of 5 different species, as detailed in Fig. 6. These collection events represent the first time each population has been sampled. It is important to document their location, bring the plant material into our collection and represent the genetics of these populations ex-situ in case the populations are lost. Due to the plants’ extremely remote locations, they may have never been sampled without this tool.

Samples were collected more than 1 km from the take-off location in an average of 8.5 min from take-off to landing. Batteries would typically be swapped between every other flight, both for the DJI M300 and the Mamba. As expected, the prototype was able to collect a variety of trees, shrubs and even small herbaceous plants. The Mamba was deployed from the road, after short hikes or even after helicopter drop-offs. It was used to collect samples on cliffs, on ridges and from other areas otherwise difficult to access on foot. The DJI M300 accessed 16 satellites on average during transit flights but only 9 satellites when sampling in close proximity to cliffs. Unfortunately, it was found that the DJI M300 only supports two concurrent GNSS constellations, instead of the four advertised, without the addition of a RTK base station.

**Figure 7:**
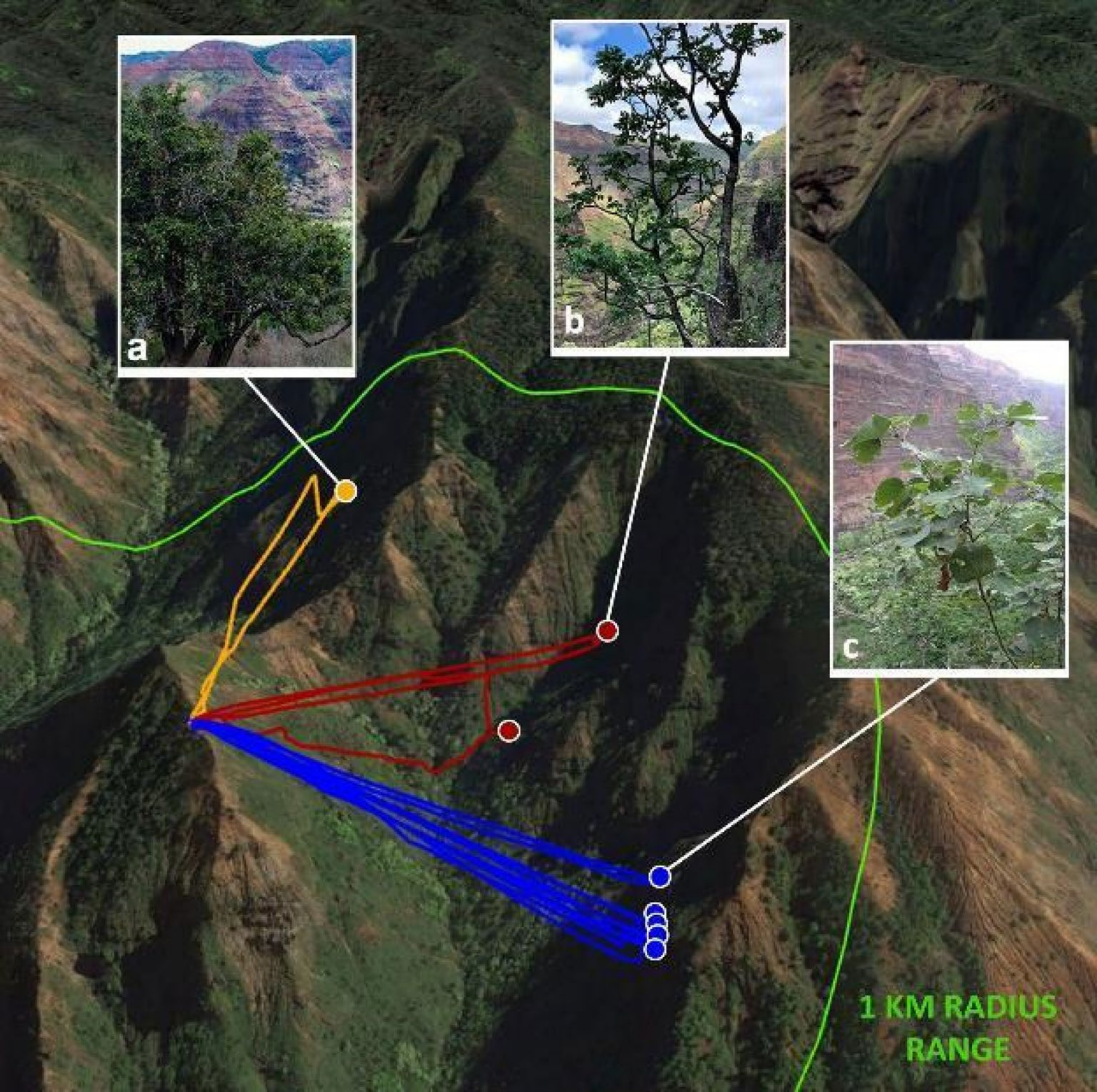
Drone trajectories during half a day of field work in the Waimea Canyon. Multiple sampling locations and species can be accessed from a single base station, in this case, (a) *Flueggea neowawraea*, (b) Polyscias *racemosa,* photo courtesy of S. Walsh, and (c) *Hibiscadelphus distans*, photo courtesy of K. Wood.

One initially unexpected advantage of the Mamba that was exploited during the final field trials resides in its capacity to rapidly access different sampling sites in a single day from a single base station. The flight paths of the fieldwork conducted on November 3, 2021 are illustrated in Fig. 7, and represent a prime example of the reach enabled by the Mamba. Plant collections were made from three distinct areas that are separated by as much as 1100 m, from a single nearby high point that is more easily accessible. This capability facilitates operational planning, increases efficiency (e.g., less packing and unpacking) and significantly reduces the cost of sampling (e.g., number of helicopter flights required to access different locations).

## Discussions

Only a few examples exist of manipulators installed on an actuated platform suspended under a drone. These systems are typically used to increase the vertical clearance between the manipulator and the drone. They are also stabilized around their point of equilibrium directly under the drone^24,25^. The suspended aerial manipulation platform presented here distinguishes itself by its long horizontal reach that keeps the lifting drone away from cliffs, its dedicated actuators and IMU-based control for increased robustness in windy and GNSS degraded conditions, as well as its specialized sampling system designed to collect different parts of a large variety of plant species. Furthermore, its performance has been demonstrated in the field through the successful sampling of 11 different critically endangered plants that would otherwise have been extremely difficult to access.

The separation of tasks between the lifting drone and the suspended platform brings numerous advantages. The cable decouples the motion of the lifting drone from the platform, as well as the contact forces and moments that the suspended platform can transmit to the lifting drone. Freed from having to fight gravity, the suspended platform can be optimized for accurate positioning in windy conditions. The suspended platform also keeps the lifting drone away from the cliff faces. When combined with basic assistance (e.g., attitude-hold on the suspended platform), this significantly reduces the mental and temporal demands needed for teleoperation. This in turn allowed a scientist with flight experience on small commercial drones (e.g., DJI Phantom 4) to collect a plant from a remote cliff without any prior training on the Mamba, indicating that the operation is intuitive enough for non-roboticists. Such easy operation of the Mamba by conservation scientists already working in the field is likely to encourage its adoption.

Overall, the field trials were highly successful. Some improvements could still enhance operations. For example, the pendulum motion of the suspended platform implies an upward motion during forward travels. This requires some coordination between both operators and/or some familiarization with the system. An additional actuator could be integrated on the cable to compensate for that vertical motion. Operations on overhanging cliffs are also currently limited by the 3.3 m effective reach of the Mamba. A longer cable could be used to increase the reach at the expense of positioning accuracy. First person view (FPV) was also used to direct the Mamba to the desired target on the cliff. However, in close proximity to the cliff, the operator could easily lose sight of the plant of interest, slowing down the operations. Using previously acquired GNSS coordinates to automatically reach the plant of interest, or an external observer further away from the cliff face (e.g., drone, telescope), would facilitate field deployment. GNSS coverage was found to be sufficient during all flights attempted, even with only two constellations. However, one site located in a tight valley with cliffs on three sides was not attempted due to GNSS coverage concerns.

Although the system has been used to collect cliffs plants for a conservation purpose, the Mamba’s versatility allows for several other applications in plant conservation. For example, during training for the field trials, several tree branches were collected. These tests revealed that the Mamba is particularly useful to sample branches on the sides of trees. The manipulator could also be easily scaled up or down to collect samples of different sizes, or be modified to enable other applications, such as placing protective bags around seed pods, collecting vegetal samples, of lichen and mosses and installing sensors or cameras. Finally, it is important to remember that, as with any tool, the Mamba could also be deployed for nefarious uses (e.g., poaching). Fortunately, the advanced skills and resources required to create such a platform should limit access to this technology in the near future.

This work describes the first aerial manipulator designed to allow scientists to sample otherwise inaccessible and critically endangered plants on cliffs. The cuttings and seeds that were collected as part of this project are currently surviving at both NTBG and State of Hawaii nurseries and will be instrumental in the *ex-situ* conservation of these species. This demonstrates that the Mamba can be used to complete the conservation life cycle for organisms located in difficult areas, from location to collection, then cultivation and outplanting. In the uphill battle against plant extinction, we hope that new robotics tools will continue to be developed and used to aid conservation efforts.

## Methods

### Hardware details

The Mamba is composed of several electrical and mechanical components, as shown in Fig. 3. The thrusters used are composed of DJI E2312 BLDC motors, E series 430 4S 30A ESCs as well as 9450 propellers. The battery used to power the entire system is a lithium polymer battery with a nominal voltage of 11.1 V and a capacity of 5200 mAh. The mechanical structure consists of a carbon rod as well as an attachment designed specifically for this project and produced using an Onyx Two Markforged 3D printer. The platform flight controller is a CUAV-X7 pro comprising an ADIS16470 tactical IMU. The RF transmission system used is a Cubepilot Herelink with a software developed specifically for this project as shown in Fig. 5.

The cutting system is controlled by a printed circuit board designed for this project. The articulated joint with two DOF is composed of two Dynamixel servo motors, a XL430-W250-T, and a XM430-W350-T. The robotic gripper used to hold the sample in place is actuated with a servo motor (Savox SA-1256TG). The robotic gripper bringing the branch to the saw is a Dynamixel servo motor (XM430-W350-T). The blade installed on the cutting mechanism motor (BadAss BA2814-1300 kV) is a 3 inches round saw.

### System evaluation

The data obtained from the tests to validate the accuracy of the Mamba in the interior flight room were recorded with a VICON system and the Matlab R2021a software version 9.10.0 (https://www.mathworks.com/products/matlab.html). During these tests, the position and orientation of the platform was acquired at a frequency of 100 Hz.

### Relevant legislations, permitting and consent

The collection of plant material, comply with relevant institutional, national, and international guidelines and legislation, IUCN Policy Statement on Research Involving Species at Risk of Extinction and Convention on the Trade in Endangered Species of Wild Fauna and Flora. These plants were collected in close coordination with the State of Hawaii staff under the Threatened and Endangered Species Permit (# I2499) and Forest Reserve Special Use Permit (#Kpi-2021-247). Voucher specimens of the plants collected have been deposited in a publicly available herbarium of the National Tropical Botanical Garden-PTBG (collector numbers: AMW 705, AMW 706, AMW 709, BN 006, BN 007, BN 011). Living material is growing at NTBG nursery (accession numbers: 20210566–2020572). Dr. David H. Lorence, PTBG Curator, undertook the taxonomic review of these plant specimens.

Field trials were conducted under the supervision of a FAA certified drone operator, and in accordance with FAA part 107.

Participants in the supplemental video work for organizations listed in the Acknowledgements section, but they are not part of the authors of this article. All participants in the supplemental video provided their informed consent to publish the information/image(s) in an online open-access publication.

## Supplementary Information

Supplementary Video S1.

Supplementary Information 2.

Supplementary Information 3.

Supplementary Information 4.

Supplementary Information 5.

Supplementary Information 6.

Supplementary Information 7.

Supplementary Information 8.

Supplementary Information 9.

## Data Availability

No datasets were generated or analyzed during the current study.
