# Author Correction: Collecting critically endangered cliff plants using a drone-based sampling manipulator

**DOI:** 10.1038/s41598-022-21945-3

**Published:** 2022-10-13

**Authors:** Hughes La Vigne, Guillaume Charron, Julien Rachiele-Tremblay, David Rancourt, Ben Nyberg, Alexis Lussier Desbiens

**Affiliations:** 1 Createk Design Lab, Interdisciplinary Institute for Technological Innovation, University of Sherbrooke, Sherbrooke, QC Canada; 2 National Tropical Botanical Garden, Kalāheo, HI USA; 3 Natural History Museum of Denmark, University of Copenhagen, Copenhagen, Denmark

Correction to: *Scientific Reports* https://doi.org/10.1038/s41598-022-17679-x, published online 13 September 2022

The Supplementary Video S1 published with this Article contained an error, where the text displayed between 0:58 second and 1:10 minute of the video incorrectly stated that the aerial tool "The Mamba" has led to a new species description. The original Supplementary Video S1 is provided below.

“This remote controlled tool has enabled rare plant collections from more than 1 km away and has led to a new species description.”

now reads:

“This remote controlled tool has enabled rare plant collections from more than 1 km away and has helped find potential new species.”

This error has now been corrected in the Supplementary Video S1 file that accompanies the original Article.

## Supplementary Information

Supplementary Video S1.

