# Supplementary figures and images for: Collecting critically endangered cliff plants using a drone-based sampling manipulator
Source: Sci Rep. 2022 Sep 13;12:14827. doi: 10.1038/s41598-022-17679-x (PMC9470584; doi:10.1038/s41598-022-17679-x)

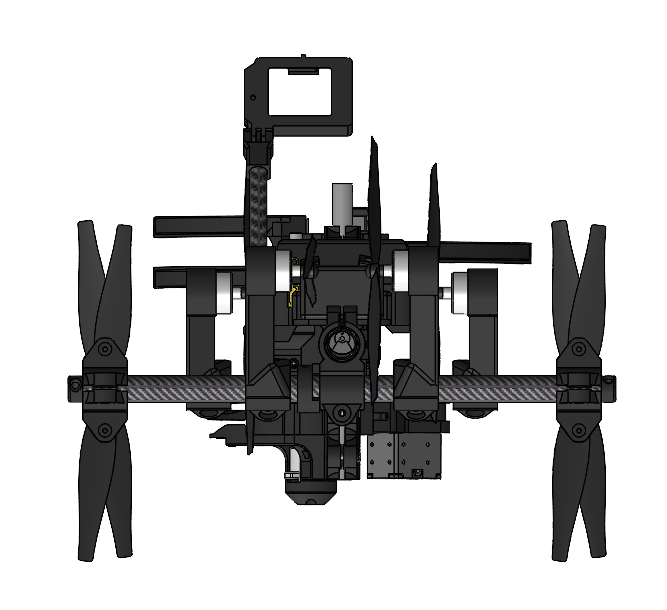

Supplement: Supplementary file 3 — Supplementary Information 3. [file 41598_2022_17679_MOESM3_ESM.png]

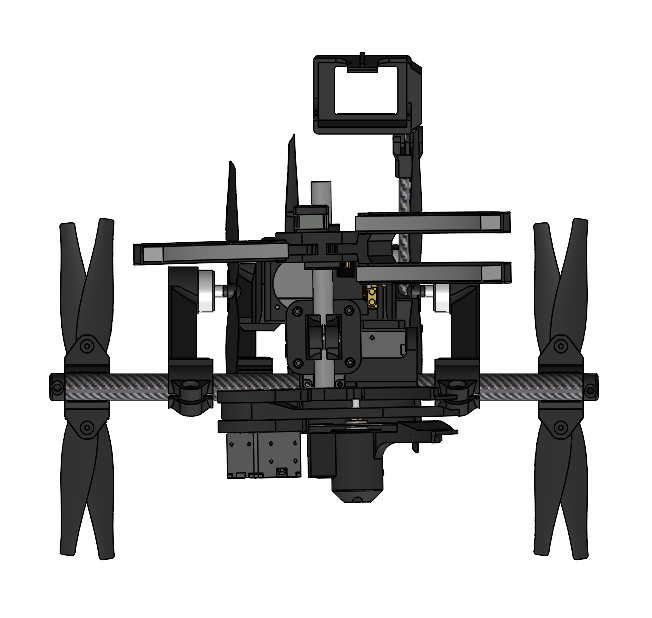

Supplement: Supplementary file 4 — Supplementary Information 4. [file 41598_2022_17679_MOESM4_ESM.png]

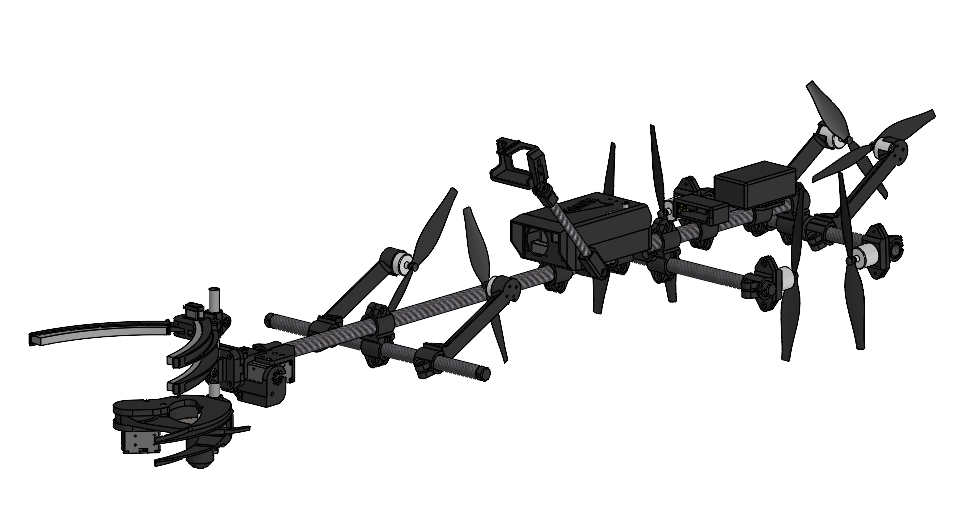

Supplement: Supplementary file 5 — Supplementary Information 5. [file 41598_2022_17679_MOESM5_ESM.png]

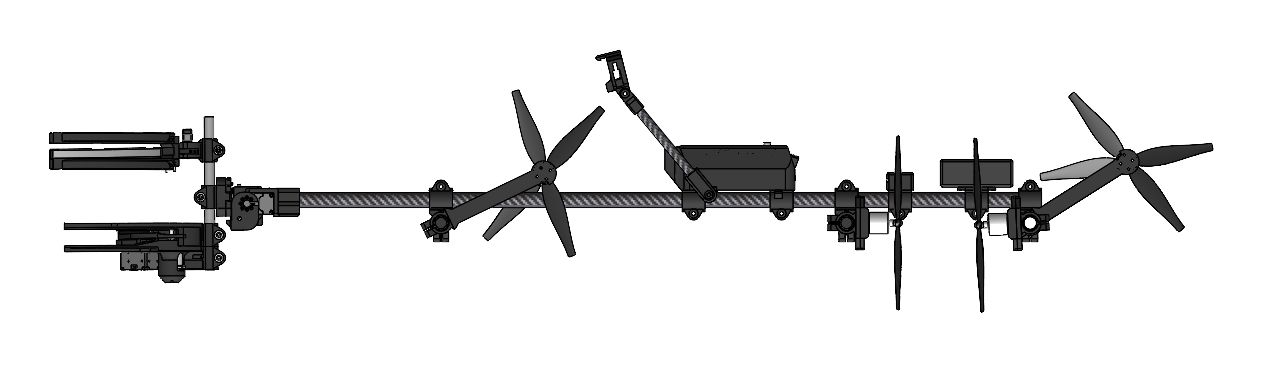

Supplement: Supplementary file 6 — Supplementary Information 6. [file 41598_2022_17679_MOESM6_ESM.png]

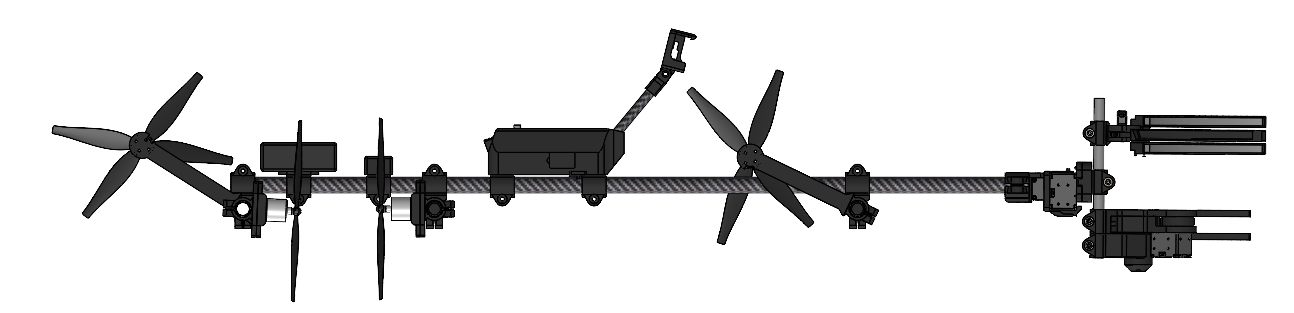

Supplement: Supplementary file 7 — Supplementary Information 7. [file 41598_2022_17679_MOESM7_ESM.png]

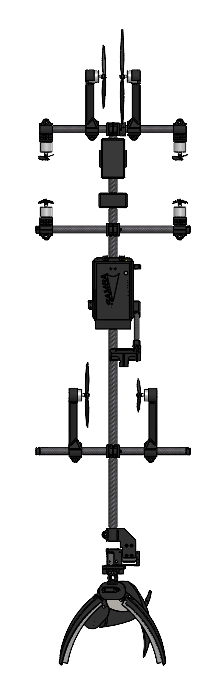

Supplement: Supplementary file 8 — Supplementary Information 8. [file 41598_2022_17679_MOESM8_ESM.png]

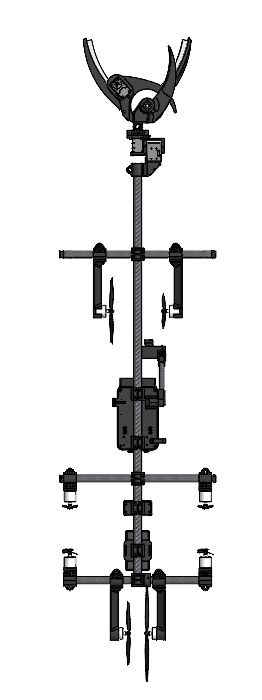

Supplement: Supplementary file 9 — Supplementary Information 9. [file 41598_2022_17679_MOESM9_ESM.png]
